# Tissue-Resident T Cells in Chronic Relapsing–Remitting Intestinal Disorders

**DOI:** 10.3390/cells10081882

**Published:** 2021-07-25

**Authors:** Juliana Barreto de Albuquerque, Christoph Mueller, Bilgi Gungor

**Affiliations:** Division of Experimental Pathology, Institute of Pathology, University of Bern, 3008 Bern, Switzerland; christoph.mueller@pathology.unibe.ch

**Keywords:** tissue-resident T cells, T cell retention, circulating T cells, memory T cells, intestinal inflammation

## Abstract

Tissue-resident memory T (T_RM_) cells critically contribute to the rapid immunoprotection and efficient immunosurveillance against pathogens, particularly in barrier tissues, but also during anti-tumor responses. However, the involvement of T_RM_ cells also in the induction and exacerbation of immunopathologies, notably in chronically relapsing auto-inflammatory disorders, is becoming increasingly recognized as a critical factor. Thus, T_RM_ cells may also represent an attractive target in the management of chronic (auto-) inflammatory disorders, including multiple sclerosis, rheumatoid arthritis, celiac disease and inflammatory bowel diseases. In this review, we focus on current concepts of T_RM_ cell biology, particularly in the intestine, and discuss recent findings on their involvement in chronic relapsing–remitting inflammatory disorders. Potential therapeutic strategies to interfere with these T_RM_ cell-mediated immunopathologies are discussed.

## 1. Introduction

Tissue-resident memory T (T_RM_) cells mediate rapid immunosurveillance and immunoprotection during reinfection with potential pathogens. They show a distinct gene expression profile, which is remarkably conserved between mouse and human T_RM_ cells [1,2], that distinguishes them from effector T cells and other memory T cell subsets. Although T_RM_ cells are readily distinguished from other T cell subsets, with the increasing availability of single-cell RNA sequencing (scRNAseq) data, the heterogeneity of T_RM_ cells in both human and mice [3,4] is becoming obvious. T_RM_ cells are the most abundant in barrier tissues, including mucosal tissues and the skin, which represent the main entrance sites for potential pathogens [5,6,7], and may also exert critical functions in T cell-mediated anti-tumor responses [8]. On the other hand, a critical involvement of T_RM_ cells in the induction and exacerbation of immunopathologies, including inflammatory bowel diseases (IBD) and celiac disease, has been clearly identified [9,10,11,12].

Chronically relapsing inflammatory diseases in humans include debilitating disorders such as multiple sclerosis, rheumatoid arthritis, celiac disease and IBD. IBD is characterized by chronic inflammation of the gastrointestinal (GI) tract and includes Crohn’s disease (CD), where any segment of the GI from the mouth to the anus can become affected; ulcerative colitis (UC), limited to the colon; and indeterminate colitis, when, at the time of diagnosis, a distinction between CD and UC is not (yet) possible [13,14]. The disease course is clinically characterized by episodes of active disease, which may become quiescent after therapy, and spontaneous relapse within months to years [15,16,17]. Current evidence suggests that inflammatory bowel diseases result from an inappropriate inflammatory immune response to intestinal microbe-derived antigens in a genetically susceptible host [18]. IBDs are chronic, lifelong diseases, which severely affect physical, psychological and social aspects of life and, thus, have a major impact on quality of life [14]. An estimated 25–40% of patients with IBD will also develop extra-intestinal manifestations during their lifetime [19]. At 30 years following the first diagnosis, 50% of patients experience at least one extra-intestinal manifestation [20]. Despite enormous efforts and considerable progress in our understanding of the pathogenic mechanisms, which also resulted in novel concepts of treating patients with IBD to allow for prolonged remission periods, there is currently still no cure for UC or CD [14]. Therefore, new candidate treatments that allow us to further delay, or even prevent, relapses of these debilitating disorders are needed. Furthermore, the insight gained into the pathophysiology of IBD may further our understanding also of the pathogenetic mechanisms operative in other chronic inflammatory diseases [21].

Celiac disease (CeD), as another relevant chronic relapsing–remitting disease of the intestine, is caused by an abnormal small intestinal T cell response to gluten, which is the major storage protein of wheat and related cereals. The disease has a strong MHC (HLA) association, and CD4^+^ T cells recognizing gluten epitopes presented by disease-associated HLA- molecules (mostly, HLA-DQ2.5, but also HLA-DQ8 or HLADQ2.2) are considered to be the critical drivers of the disease [22]. Originally, CeD was believed to represent a rare malabsorption syndrome in children and teenagers. However, it is now increasingly recognized as a common condition that may be diagnosed at any age.

A gluten-free diet is key for a successful therapy; however, relapse of the disease is seen in a considerable fraction (up to 30%) of CeD patients, mainly due to dietary non-adherence [23]. In contrast to the huge variety of intestinal lumen-derived microbial antigens as triggers of the disease in IBD, in CeD, the triggering antigen, i.e., the dietary protein gluten, modified by the host-derived enzyme tissue transglutaminase, is well characterized [24].

This review focuses on the biology of T_RM_ cells, with special emphasis on their involvement in chronic intestinal inflammation. Furthermore, we also discuss their potential as targets in the management of chronic relapsing–remitting inflammatory disorders, particularly when T_RM_ cell-specific pathways can be manipulated.

## 2. Tissue-Resident Memory T Cell Subsets in the Intestine

**Figure 1:**
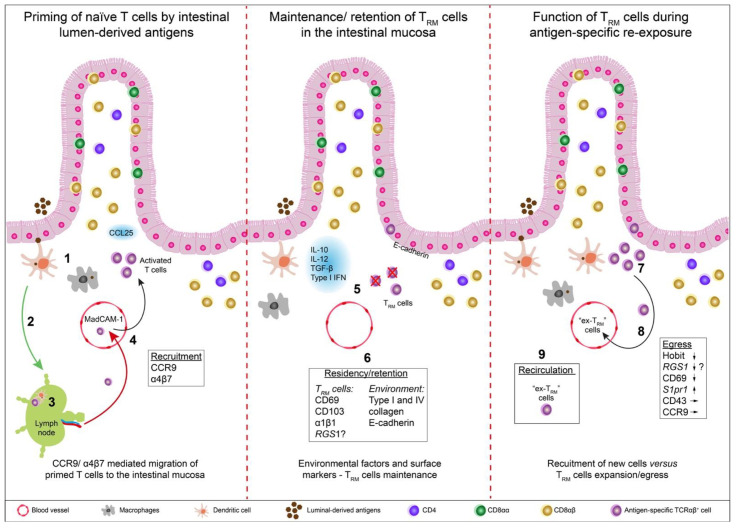
Activation of conventional intestinal T cells following primary exposure to a novel, MHC-restricted antigen versus re-exposure of T_RM_ cells to their cognate antigen. (**1**) Intestinal lumen-derived antigens (microbiota, food antigens, pathogens) breach the intestinal epithelial barrier through specialized M cells overlying intestinal Peyer’s patches and solitary follicles (not shown), or obtain access to the intestinal lamina propria when the integrity of the epithelial layer is disrupted. Inside the lamina propria, antigens are rapidly taken up and digested by local macrophages, which are rather poor antigen-presenting cells. A fraction of the intestinal lumen-derived antigens, however, is sampled by intestinal dendritic cells. (**2**) These antigen-loaded dendritic cells become activated and migrate to the draining mesenteric lymph nodes, (**3**) where they present the antigen in an MHC-restricted manner to antigen-specific naïve CCR7^+^ CD62L^+^ T cells. (**4**) Upon their priming in the draining lymph nodes, these activated T cells acquire a gut homing phenotype, characterized by the cell surface expression of α4β7 and CCR9, which bind to MadCAM-1 expressed on gut-associated endothelial cells, and to the chemokine CCL25, secreted by endothelial cells in the small intestine and by follicle-associated epithelium of Peyer’s patches, respectively. This imprinted phenotype allows the primed T cells to home back to the site of initial antigen breaching. (**5**) Under the influence of the local intestinal microenvironment (e.g., TGF-β, IL-12 and type-I IFN), some of these recently activated T cells acquire a T_RM_ cell signature. (**6**) Within the intestinal mucosa, transcription of the α4 chain gene is suppressed in T cells. TGF-β, secreted by macrophages and dendritic cells, induces the expression of αE integrin (CD103). The αEβ7 heterodimer binds to E-cadherin, which is expressed on epithelial cells and subsets of dendritic cells. Subsets of CD8^+^ T_RM_ cells also express the integrin α1 chain together with the β1 integrin (α1β1), which directly binds to type I and IV collagen and, hence, supports the adherence and retention of T_RM_ cells in skin, lung and intestine. Most T_RM_ cells also express CD69, which antagonizes S1P_1_ (Sphingosine 1 phosphate receptor-1), thus contributing to their retention within the tissue. RGS1, which attenuates signaling via Gαi- or Gαq- linked G-protein-coupled receptors, is also highly expressed in T_RM_ cells. This signature allows them to remain at this site even upon complete clearance of their cognate antigen. These newly generated T_RM_ cells will thus continuously expand the existing repertoire of antigen specificities of the local T_RM_ cells. Some of these activated T_RM_ cells (mostly CD8αβ^+^ TCRαβ^+^, CD8αα^+^ TCRαβ^+^ and TCRγδ^+^ cells) will home to the intestinal epithelium (“intraepithelial lymphocytes”), where they are preferentially retained by the interaction of the αEβ7 integrin on their surface with epithelial cell-expressed E-cadherin. (**7**) Upon re-exposure to the cognate antigen, T_RM_ cells are rapidly activated and expand locally in the mucosa. They are prone to efficiently secrete cytokines and express cell surface molecules, which may further enhance the uptake and degradation of incoming microbes or dietary compounds by local (resident) macrophages. Reactivated T_RM_ cells may express cytotoxic effector molecules, including granzymes and perforin, or secrete chemokines that promote the recruitment of other leukocyte subsets, including monocytes, but also effector T cells. (**8**) Additionally they can loose some T_RM_ cell markers and retention profile (Hobit, CD69, and potentially *RGS1*) and upregulate genes related to egress (*S1pr1*). (**9**) Thus, eventually, they can re-enter the blood circulation as “ex-T_RM_” cells, becoming circulating effector cells and memory T cells, thus, supporting also the systemic immune response.

Naïve T cells are activated in intestine-draining lymph nodes by antigen-loaded dendritic cells that migrate to these sites following antigen uptake in the intestinal mucosa. Most of these activated T cells are instructed by the local environment to express tissue-specific chemokine receptors, notably CCR9, to migrate back to the site of antigen entry [25]. In the local microenvironment of the intestinal mucosa, they may subsequently differentiate into local T_RM_ cells to become an integral part of the immune sensing network. They monitor the tissues for local perturbations in homeostasis and are involved in the immunosurveillance against infection and cancer, but are increasingly recognized as promotors of immunopathologies [26]. During a local reinfection, T_RM_ cells rapidly exert their immune functions, such as cytotoxic effector functions, or the secretion of chemokines, to amplify locally the adaptive and innate immune response against pathogens, thus critically contributing to immunosurveillance and immunoprotection [27,28,29] (Figure 1).

In the intestinal mucosa, T_RM_ cells are found in both the intestinal epithelium (“intraepithelial lymphocytes”, IEL) and in the lamina propria (“lamina propria lymphocytes”, LPL). These two compartments are only separated by the basement membrane; their cellular composition, however, differs considerably. In the mouse intestine, CD8αα^+^ and CD8αβ^+^ TCRαβ^+^ T cells, together with TcRγδ^+^ T cells, dominate in the IEL compartment [30]. CD8αα^+^ TCRαβ^+^ T cells and TCRγδ^+^ T cells are considered unconventional IEL [31]. In contrast to conventional CD4^+^ and CD8αβ^+^ TCRαβ^+^ T cells, in the mouse, the unconventional CD8αα^+^ TCRαβ^+^ T cells and TCRγδ^+^ T cells do not undergo an intrathymic negative selection [32] and may leave the thymus before this differentiation program is initiated. At a transcriptional (and functional) level, intestinal CD8αα^+^ TCRαβ^+^ IEL are thus more related to TCRγδ^+^ IEL than to CD8αβ^+^ TCRαβ^+^ IEL [33].

In the intestinal lamina propria, the distribution of T cells more closely resembles the composition of lymphoid organs. CD4^+^ T cells represent the predominant intestinal lamina propria T cell subset in both the small and large intestine, unconventional CD8αα^+^ TCRαβ^+^ T cells are absent, and TCRγδ^+^ T cells are present only at lower frequencies than in the IEL compartment [34,35]. Gut-associated lymphoid tissues, such as Peyer’s patches, colonic patches and solitary lymphoid follicles, are embedded within the intestinal lamina propria and form organized induction sites together with the draining lymph nodes, which act as an immunological firewall for intestinal lumen-derived antigens to allow for an efficient adaptive immune response [36,37].

## 3. Differentiation of T Cells after Initial Antigen Exposure

CD8^+^ T cells expand and differentiate upon initial antigen-specific priming into phenotypically distinct subsets. Early after antigen-exposure, early effector T cells (EECs) dominate the initial antigen-specific T cell response. EECs lack cell surface expression of the IL-7 receptor-α chain (CD127) and of the killer lectin-like receptor 1 (KLRG1). Intriguingly, a single naïve CD8 T cell may give rise to different fates, and may also further differentiate into memory precursor effector cells (MPECs), characterized by a CD127^+^ KLRG1^−^ phenotype, and CD127^−^ KLRG1^+^ short-lived effector cells (SLECs) [38,39]. SLECs are responsible for the rapid, high-affinity CD8^+^ T cell-mediated immune response [40,41]. On the other hand, KLRG1^−^CD127^+^ MPECs further differentiate into circulating memory and resident memory T cells [42]. TCR affinity appears to regulate the generation of MPECs versus SLECs: intriguingly, T cells with a high-affinity TCR appear to be prone to develop into SLECs, while T cells with an intermediate-affinity TCR preferentially differentiate into MPECs, possibly reflecting a “trade-off” between the rapid expansion of high-affinity T cells versus the long-term production of intermediate-affinity T cells [43,44]. In an acute influenza infection model, CD8^+^ T cells with a high-affinity TCR were more prone to differentiate into SLECs, while T cells with a lower-affinity TCR in this mouse model preferentially differentiated into CD8^+^ T_RM_ cells or CD8^+^ T_EM_ cells [45]. Extrinsic factors may also direct the fate of CD8^+^ T cells even before initial cognate antigen recognition: TGF-β produced by migratory dendritic cells in draining lymph nodes can epigenetically condition naïve CD8 T cells to differentiate into epidermal T_RM_ cells upon intradermal vaccination with a plasmid-encoding chicken ovalbumin [46]. Accordingly, the route of pathogen entry also appears to influence the homing of the precursors and T_RM_ cell differentiation. For instance, CD8^+^ T_RM_ cells in the intestinal epithelium were activated by oral infection with *Listeria monocytogenes*, but not by infection via the intranasal or intravenous routes [47]. Hence, the local cytokine milieu present at the site of antigen priming appears to be a critical factor to direct the generation of T_RM_ cells in barrier tissues. In this respect, the cytokines TGF-β, IL-15, IL-12 and type-I IFN are critical in the early phase of T_RM_ cell differentiation [1,48] (Figure 1).

## 4. Retention of T_RM_ Cells in the Intestinal Mucosa

T_RM_ cells were operationally identified in parabiosis experiments, where, in contrast to the circulating T cell subsets, T_RM_ cells were not shared among the two parabiotic partners in short-term parabiosis experiments [49,50,51,52]. The development and functional differentiation of T_RM_ cells includes several checkpoints, including the entry into their target tissues, local retention and subsequent responsiveness to local cytokines and other factors that support T_RM_ cell formation and survival [53].

Hence, it is not surprising that the transcriptional signatures of T_RM_ cells in humans and in mice are distinct from naïve T cells, T_CM_, and T_EM_ cells. The core T_RM_ cell signatures are remarkably consistent in T_RM_ cells from different barrier tissues in mice and humans [2,54,55,56], although functional heterogeneities are increasingly observed among T_RM_ cell populations within the same tissue, particularly upon scRNAseq analyses [4,55]. Most of these T_RM_ cell signature genes encode proteins with a direct, or indirect, involvement in the retention and prolonged maintenance in the tissue as long-lived T_RM_ cells [42]. Furthermore, T_RM_ cells are also characterized by the expression of genes (e.g., *Bhlhe40*) which enhance their metabolism by conferring increased mitochondrial fitness, while the expression of distinct genes which are overexpressed in T_CM_, T_EM_, or T_EFF_ cells is completely absent in T_RM_ cells, such as KLRG1, CCR7, S1P_1_ and KLF2 (Table 1).

In humans and mice the recruitment of circulating T cell subsets to mucosal sites is directed by the integrin α4β7, which becomes expressed in EEC subsets upon antigen-specific activation at inductive sites. This integrin binds to the mucosal addressin MadCAM-1, and, hence, is most critical for the recruitment of T cells to mucosal sites. Upon diapedesis of α4β7-expressing T cells into mucosal sites, the transcription of the *Itga4* gene is suppressed and the expression of *Itgae* is induced by bioactive TGF-β, which is present locally at elevated levels, resulting in an exchange of the α4-integrin chain by the αE integrin (CD103). The αEβ7 heterodimer binds to E-cadherin, which is expressed on epithelial cells [72], but also on haematopoietic cells, including inflammatory dendritic cells in draining lymph nodes [73]. CD103 expression is more prominent in CD8^+^ T_RM_ cells than in CD4^+^ T_RM_ cells in both humans and mice [62]. Oral infection studies with *Listeria monocytogenes* revealed that CD103 is indeed essential for the local accumulation of the CD8^+^ T cells in the small intestine epithelium and LP, yet, is dispensable for T cell retention [29,47]. However, also CD103^−^ CD8^+^ T_RM_ cells were found, notably in the intestinal lamina propria after *Yersinia pestis* infection [62], and the size of this CD103^−^CD8^+^ T_RM_ cell population appears to be TGF-β-independent, but is critically regulated by IFN-γ and IL-12 [48]. Subsets of CD8^+^ T_RM_ cells also express the integrin α1 chain (CD49a), together with the β1 integrin (α1β1), which directly binds collagen type I and IV and, hence, supporting the adherence and retention of T_RM_ cells in the skin, lung and intestine [64,74] (Table 1, Figure 1).

Sphingosine-1-phosphate receptor (S1P_1_) is a critical checkpoint for T_RM_ cell persistence [27]. It facilitates T cell exit out of lymph nodes in response to higher S1P levels within the efferent lymph [75]. S1P_1_ expression is regulated by the transcription factor Kruppel-like factor 2 (KLF2), and S1P_1_ signaling is antagonized by CD69, a type II-C lectin receptor [61], which is part of the T_RM_ cell signature, but is also transiently upregulated on T cells upon antigen-specific activation [76]. Similar to T_RM_ cells from other mucosal tissues, intestinal T_RM_ cells decrease the expression of KLF2 and upregulate CD69 upon migration to their site of residence. This differential expression of KLF2 and CD69 suppresses S1P_1_ responsiveness and restrains the T_RM_ cells in the tissues [77]. Although CD69 is commonly believed to represent a surrogate marker for the identification of T_RM_ cells, some recent reports suggest that CD69 is dispensable for T_RM_ cell formation and maintenance in some barrier tissues, including the small intestine, lung and female reproductive tract [78]. Moreover, deletion of CD69 in mice does not affect CD4^+^ T_RM_ cell formation, while the frequencies and numbers of CD8^+^ T_RM_ cells in the skin and lung were reduced after influenza virus infection in CD69-deficient mice, indicating a differential requirement for CD69 for the retention of CD4^+^ versus CD8αβ^+^ T cells, which may further be influenced by the site of residency [42].

Similar to S1P_1_, the chemokine receptor CCR7 is upregulated by KLF2 and downregulated in T_RM_ cells. It guides mostly naïve T cells along a CCL21 and CCL19 gradient into the T cell zone of secondary lymphoid organs. The absence of CCR7 expression on T_RM_ cells likely prevents their migration out of the intestinal mucosa to the CCL19 and/or CCL21 containing afferent lymphatics [61]. Furthermore, the gut homing receptors CCR6 and CCR9 are upregulated in T_RM_ cells to retain both CD4^+^ and CD8^+^ T cells at these mucosal sites, with elevated levels of their corresponding ligands CCL20 (expressed in the small intestine) and CCL25 (expressed in the small and large intestine), respectively [2,4,79,80]. Hence, also the responsiveness of T cells to chemokines in barrier tissues (and the mode of their regulation) is critical for defining the retention of T_RM_ cells in the tissue. Some members of the the regulator of G protein signaling (RGS) family, including RGS1, are consistently overexpressed in T_RM_ cells from barrier tissues [65,81,82,83], but also in tumor-infiltrating T cells [84,85]. These RGS family members enhance the GTPase activity of GTP-bound Gαi (also Gαq) and, thus, inhibit Gαi/αq-coupled receptor signaling [86,87]. Chemokine receptors are all members of the Gαi family of G-protein linked receptors (GPCR); thus, RGS1 is considered a potential repressor of chemokine-mediated T cell egress and supports local T_RM_ cells retention in the gut. The capacity of RGS1 to regulate directional chemotaxis was confirmed in trans-well experiments with CCR7 and CXCR4 expressing Jurkat cells, which, upon transfection with the *RGS1* gene, displayed impaired migration to the lymph node chemokines CCL19 (ligand for CCR7) and CXCL12 (ligand for CXCR4) [65].

The local cytokine microenvironment in the intestinal mucosa critically shapes intestinal T_RM_ cell differentiation and tissue retention. Bioactive TGF-β is present in many epithelial surfaces, including the small and large intestinal epithelium, and promotes CD103 expression on T_RM_ precursor cells, which allows the enhanced retention of T_RM_ cells in e-cadherin-expressing epithelia [29,42,47]. TGF-β is produced and secreted as a biologically inactive precursor bound to the latency associate peptide. This biologically inactive complex is stored in the extracellular matrix before it is processed and cleaved to release its biologically active form of TGF-β. This is achieved by integrin-dependent and -independent mechanisms. The extracellular processing of the latent TGF-β complex represents a key element in the regulation of TGF-β activity. Integrin-independent mechanisms of TGF-β processing may include proteolytic cleavage, lower pH and thrombospondin-induced cleavage, while αVβ6 and αVβ8 integrins are responsible for integrin-mediated TGF-β processing [88]. The preferential expression of these integrins in epithelia may thus represent a critical factor for controlling the retention of local resident cell subsets in epithelial tissues [89]. Indeed, in the skin, competition for active TGF-β allows the selective retention of antigen-specific T_RM_ cells in the epidermal niche [90].

Downregulation of both T-box transcription factors T-bet/*Tbx21* and *Eomes* represents—at least in the skin—a critical step in the local differentiation of CD8^+^ CD103^+^ T_RM_ cells. TGF-β-signaling downregulates the expression of these T-box transcription factors, while, conversely, T-bet and Eomes downregulation are a prerequisite for TGF-β cytokine signaling. In the complete absence of Eomes expression, however, skin CD8^+^ CD103^+^ T cells become dependent on residual T-bet expression, which controls the expression of CD122 (IL-15RB), and hence the responsiveness of T_RM_ cells to IL-15. IL-15 signaling appears to be critical for the survival and maintenance of T_RM_ cells in the skin, lungs, liver, salivary glands and kidney, whereas in the small intestinal mucosa of mice, T_RM_ cells proliferate and persist even in the absence of IL-15 [1,91]. In mice, the transcription factor “homolog of blimp1” (Hobit), encoded by the *Znf683* gene and Blimp1, encoded by the *Prdm1* gene, control the expression of CD69, KLF2 and S1P_1_, which are critically required for CD8^+^ and CD4^+^ T_RM_ cell development and persistence in most barrier tissues in mice, including the intestine [9,54]. In humans, however, Hobit is not differentially expressed in T_RM_ cells versus circulating CD8^+^ T cells and, thus, may not be specific for T_RM_ cells [92]. Runx3 is a central regulator of T_RM_ differentiation and supports the expression of genes related to tissue residence, such as *Cd69* and *Itgae*, but also *Gzmb*, while suppressing the expression of genes involved in cell egress, including *Klf2*, *S1pr1* and *Ccr7 [60]*. The differential expression of the two transcription factors Blimp1 and Id3 was used to identify the functional diversity within the SI IEL CD8^+^ T cells, induced by an LCMV infection in mice [55]. Early after LCMV infection, Blimp1 expression was increased in KLRG1^hi/int^CD127^lo^ cells, which also showed the expression of genes associated with effector functions of T_RM_ cells, such as elevated levels of granzyme B. On the other hand, KLRG1^lo^CD127^hi^ small intestinal CD8^+^ T cells expressed high levels of Id3 rather than Blimp1 and they shared their transcriptional signatures with the other memory, or memory-like, cells, including T_CM_ and T_FH_ cells in the effector phase and T_RM_ cells in the memory phase of infection. Id3^hi^ Blimp^lo^KLRG1^lo^CD127^hi^ had enhanced memory potential and, upon recall infection, they rapidly proliferated and generated a great frequency of resident and re-circulating cells [55].

The development of T_RM_ cells in barrier tissues is also affected by dietary components. As an example, the aryl hydrocarbon receptor (Ahr) is an essential regulator in maintaining IEL numbers in the skin [93] and the intestine [66]. Intestinal Ahr signaling is regulated by dietary products (e.g. cruciferous vegetable-derived products, glucosinolate glucobrassicin I3C) and environmental pollutants, but also by microbiota-derived Trp-catabolites that act as an Ahr ligand (indole-3-aldehyde, indole acrylic acid, indole acetic acid or tryptamine from *L. reuteri* and many other Firmicutes including members of the *Clostridium* genus) [66,94,95]. In the intestinal mucosa Ahr signaling appears to be critical for maintaining an intact epithelial barrier, including the retention of intestinal IEL, notably TCRγδ^+^ T cells. As a consequence, the absence of Ahr ligands causes a change in the luminal microbial load and composition, and increased signs of immunopathological changes in the epithelium [66]. Furthermore, Ahr-deficient mice develop exacerbated dextran-sodium sulfate (DSS)-induced colitis with elevated expression of pro-inflammatory cytokine genes (e.g., IL-1β, IL-6, TNF-α, when compared to DSS-treated, Ahr-sufficient wildtype mice [96]. Intriguingly, in patients with IBD, particularly in patients with CD, AhR signaling in intestinal tissues is downregulated [97].

Due to their preferential localization within the barrier tissues, T_RM_ cells generally have restricted access to nutrients and oxygen compared to circulating T cells. Thus, T_RM_ cells generally live under rather stressful conditions and, hence, show a series of particular metabolic adaptations—for example, fatty acid oxidation—to survive and function in the tissues. For this purpose, T_RM_ cells express organ-specific isoforms of fatty acid-binding proteins (FABP) for the selective uptake of fatty acids. Small intestinal CD8^+^ T_RM_ IEL express fabp1, fabp2 and fabp6, but very low fabp4 and fabp5, known to be expressed by skin T_RM_ cells [67,98,99].

The enhanced mitochondrial fitness and functions seen in T_RM_ cells contribute to their maintenance and effector cell activity. As an example, the transcription factor Bhlhe40 was shown to maintain the mitochondrial fitness and metabolism of T_RM_ cells and tumor infiltrating cells (TIL), where the expression of Bhlhe40 is selectively upregulated [42,67]. This promotes an active chromatin state for T_RM_ cell and TIL residency and functionality. The enhanced mitochondrial fitness may help to overcome the local stress factors present in the microenvironment (e.g., limited access to nutrients, such as glucose) under hypoxic or oxidative conditions [100]. The purinergic receptor P2RX7 is required for the establishment, maintenance and functionality of long-lived tissue-resident and central memory CD8^+^ T cell populations in mice. These receptors promote mitochondrial homeostasis and metabolic function in differentiating memory CD8^+^ T cells, at least in part by inducing AMP-activated protein kinase, which activates glucose and fatty acid uptake and oxidation when cellular energy is low, as is the case for T_RM_ cells in barrier tissues [101]. P2RX7-expressing cells, however, are also highly susceptible to extracellular ATP (eATP) and NAD-induced cell death (NICD) [69], which becomes particularly evident upon ex vivo isolation of these cell populations from solid tissues, where the released NAD and eATP will lead to their underrepresentation due to NICD during subsequent adoptive cell transfer, or extended in vitro cell cultures. This likely results in an underestimation of their role in systemic immunoprotection, as assessed by adoptive transfer experiments, where—in the absence of ARTC2.2-specific nanobodies during the ex vivo isolation procedure—an overwhelming portion of transferred cells will undergo rapid NAD-induced cell death [69,102] (Ch. Mueller and Leslie Saurer, unpublished observations). Hence, some of the T_RM_ cell transfer experiments reported in the literature where NICD was not blocked may need to be repeated to fully reveal the potential of T_RM_ cell-mediated immunoprotection also in- and outside their site of residence.

## 5. T_RM_ Cell Dynamics during Infection

As outlined above, T_RM_ cells, at least under homeostatic conditions, do not generally recirculate and reside preferentially in nonlymphoid tissues, particularly at barrier sites, but occasionally also in lymph nodes and the spleen or local vascular compartments [103]. The most recent data obtained with Hobit fate mapping mice [104], skin transplant experiments [105] and with long-term parabionts [106] reveal a distinct view of the biology and the impact of T_RM_ cells also for systemic immune responses. Immunosurveillance by CD8^+^ T_RM_ cells is mostly ascribed to the rapid elimination of infected cells either via cytotoxic activity by producing cytotoxic granule-associated proteins such as granzymes and perforin or by directly, or indirectly, recruiting effector cells via chemokines or inflammatory cytokines [2,26]. Moreover, skin and intestinal infection models revealed that CD8^+^ T_RM_ cells can exit peripheral tissues and recirculate during a secondary T cell response, as so-called “ex-T_RM_” cells (Figure 1). This egress of reactivated T cells from nonlymphoid tissues is partially mediated by S1P, since FTY720 treatment reduced the number of CD69^+^ T_RM_ cells in the draining lymph nodes [104,105]. In a fate mapping mouse line, Hobit reporter tdT-positive cells also expressed YFP expression, and once Hobit expression was lost, YFP remained, allowing the identification of ex-Hobit^+^ cells, which are “ex-T_RM_“ cells [104]. Upon intestinal reinfection, Hobit^+^ CD8^+^ T_RM_ cells are able to expand in the peripheral tissue and draining lymph nodes, and recirculate. These “ex-T_RM_” cells have decreased Hobit expression and acquire a T_EM_ phenotype, mostly CD62L^−^KLRG1^+^CX3CR1^+^. Downregulation of Hobit may directly influence T_RM_ cell egress and differentiation into circulating cells, as these cells lack the expression of tissue retention molecules (for example, CD69) and express *Klf2* and *S1pr1*, which support tissue egress [104]. After skin transplantation from ovalbumin-expressing vesicular stomatitis virus infected mice and local reactivation by a SIINFEKL peptide delivered by a tattoo gun, T_RM_ cells also egress from the skin into the circulation. At the transcriptome level, intestinal T_RM_ cells show a signature distinct from T_CM_ and T_EM_ cells, indicating that they are indeed functionally separate cell populations, although they share some epigenetics signatures. In a multipotency scoring system, ranging from 0 (exhausted CD8^+^ T cells) to 1 (naïve CD8^+^ cells), the plasticity score of T_RM_ cells was between the scores for T_CM_ and T_EM_ cells, suggesting that T_RM_ cells are indeed not fully differentiated yet and retain a certain level of plasticity [105]. Hence, upon antigen-specific reactivation, “ex-T_RM_” cells can differentiate into T_CM_ and T_EM_ cells, but can also generate further T_RM_ cells, thus contributing to a systemic immune response [82,105]. Upon repeated antigen exposure, e.g., following local reinfection with the same pathogen, also “ex-T_RM_” cells are observed, with downregulated CD69 and CD103 on their cell surface, however maintaining most of the T cell signature of their tissue of origin (CCR9 in intestinal T_RM_ cells) [105].

In contrast to the other studies mentioned above, extended parabiosis experiments showed the presence of circulating “ex-T_RM_” cells after primary infection. During 30 days of parabiosis, donor and host cells were equilibrated in blood, and 200 days following their physical separation, a higher percentage of host cells than donor cells was found in the peripheral blood, indicating that they originated from cells that were not able to equilibrate during parabiosis, most likely cells within the tissues (T_RM_ cells) that later rejoined the circulation (“ex-T_RM_” cells), giving rise to memory cells in blood (Figure 1). Collectively, it appears that T cells which adapted a T_RM_ cell lifestyle within non-lymphoid organs, rather than circulating adaptive immune cells, represent the main cellular players for the maintenance of local immunosurveillance, while *de novo* haematopoiesis still contributes to systemic immunosurveillance, but becomes of decreasing relevance in this process of progressive decentralization of the maintenance at the level of the organism [106]. This shift in the relevance of primary versus secondary and tertiary lymphoid tissues with age is also reflected by the sharp decline in the thymic output of T cells after puberty [107].

Most of these T_RM_ cell - and “ex-T_RM_” cell concepts were initially established in mouse models of infections; however, they have been also described in other disease models. 

## 6. T_RM_ Cells in Intestinal Inflammation

After pathogen clearance following acute infection, antigen-specific T_RM_ cells persist locally and can rapidly eliminate the pathogen upon reinfection. Unlike in acute infections, in chronic inflammatory diseases of the intestinal tract the triggering antigens (e.g., luminal microbiota- or diet-derived antigens) can persist and lead to chronic or intermittent stimulation of the local immune system, notably of antigen-specific T_RM_ cells. In recent years, evidence has been increasing for a critical contribution of T_RM_ cells in the pathogenesis of intestinal relapsing–remitting inflammatory disorders, including CeD and IBD. These diseases may be triggered by the presence of the T cell-activating antigens in the absence of appropriate immunoregulatory mechanisms, or intact epithelial barriers. In CeD, the same antigens, i.e., gluten-derived peptides, and hence also the same long-lived disease-causing T cell clones, are involved [12]. On the other hand, in IBD, the relative abundance of disease-triggering antigens may substantially differ in the different phases of remission and relapsing disease, as evidenced by the result of an extensive 16S sequencing of intestinal mucosa-associated bacteria in patients with active and inactive IBD [108].

Intriguingly, in CeD patients, circulating gluten-specific CD4^+^ T cells with a unique cell surface signature (CD38^+^, CD39^+^, CXCR3^+^, PD-1^+^, ICOS^+^, CD161^+^, CCR5^+^ and CD28^+^) were observed to show a high resemblance to gluten-specific CD4^+^ T cells found in the affected small intestinal mucosa, but also to CD4^+^ T cells in patients with systemic lupus erythematosus [109]. This distinct cell-surface signature of circulating autoinflammatory CD4^+^ T cells also overlaps with the signature previously described in patients with rheumatoid arthritis [110]. This strongly suggests that there is indeed a distinct, but rather rare, subset of CD4^+^ T cells which is common to several CD4^+^ T cell-dependent autoinflammatory disorders. Whether these circulating gluten-specific CD4^+^ T cells indeed represent circulating “ex-T_RM_ cells” which originated in the small intestinal mucosa of patients with CeD remains open. Likewise, it remains to be seen whether the appearance of circulating cells with this distinct cell surface signature can be used to predict imminent flares of chronic relapsing–remitting disorders.

In CeD, the main trigger of active disease is the dietary gluten exposure; thus, a continued gluten-free diet represents an effective treatment. Although gluten-specific CD4^+^ T cell clonotypes decrease following gluten avoidance therapy, some gluten-specific CD4^+^ T cells still persist for decades in the intestine and blood. These long-lived gluten-specific CD4^+^ T cells can be reactivated by re-exposure to even traces of gluten [12]. Further corroborating these findings on the longevity of CD4^+^ T cells in the small intestine, Bartolomé-Casado and collaborators have shown that in the human transplanted small intestine, both CD8^+^ and CD4^+^ T_RM_ cells persist in the tissue for more than 1 year. After their in vitro restimulation, a large proportion of lamina propria CD103^+^ CD8^+^ T cells produced granzyme B, perforin and one or more of the cytokines tested (IFN-γ, IL-2, TNF-α), outnumbering granzyme B, perforin and inflammatory cytokine-expressing T cells in the other CD8^+^ T cell subsets (CD103^+^ IEL and CD103^−^ LPL). Similarly, upon restimulation in vitro, small intestinal CD103^+^ CD4^+^ T cells also produced granzyme B and displayed a polyfunctional T_H_1 cell profile, while a minor proportion of CD103^+^ CD4^+^ T cells produced IL-17 [57,58].

In IBD, both CD4^+^ and CD8^+^ T cells can be associated with the induction and progression of the disease. In colonic samples from UC and CD patients, increased frequencies of CD103^+^CD69^+^ cells are seen, which show higher expression of pro-inflammatory genes (*Ifng*, *Il13*, *Il17A*, *Tnfa*) than colonic CD69^−^ T cells. Moreover, both CD4^+^CD69^+^ and CD8^+^CD69^+^ T_RM_ cells were enriched in these biopsies. Elevated frequencies of phenotypic CD4^+^ T_RM_ cells were associated with shorter flare-free intervals, while such an association was not found for phenotypic CD8^+^ T_RM_ cells [9].

In the T cell transfer mouse model of colitis, the transfer of CD4^+^ T cells lacking both Hobit and Blimp-1, which are two transcription factors associated with mouse T_RM_ cells, into lymphopenic Rag2^−/−^ mice was not able to induce colitis to the same extent as with wildtype CD4^+^ T cell transfer. Furthermore, mice that received Hobit/Blimp-1 double knockout CD4^+^ T cells showed reduced leukocyte recruitment and attenuated expression of pro-inflammatory cytokines. Hobit/Blimp-1 double knockout mice are also protected from acute TNBS-induced colitis and acute and chronic DSS-induced colitis. Additionally, DTR-mediated ablation of transferred Hobit^+^ CD4^+^ T cells protected recipient mice from colitis induction [9]. In the colon of CD patients, CD69^+^CCR7^+^ CD4^+^ T_RM_ cells are enriched and produce higher levels of IL-17A and TNF-α than controls [111]. In line with the fact that the microbiota can be involved in the pathogenesis of IBD, colonic-resident CD4^+^ T cells reactive to enteric bacteria presented increased IL-17A production in both CD and UC patients regardless of disease activity or therapy [11]. Moreover, we recently established a mouse model of reversible CD4^+^ T cell transfer colitis, which allowed us to study the role of CD4^+^ T cells in disease onset. In this model, remission could be induced by systemic anti-CD4 mAb treatment, depleting the circulating CD4^+^ T cells, and after treatment stopped, mice relapsed spontaneously, indicating a potential role of tissue-resident CD4^+^ T_RM_ cells not only in colitis induction but also in relapsing disease, which may have escaped anti-CD4 mAb-mediated depletion [112,113].

Single-cell RNA and TCR sequencing analyses of CD8^+^ T_RM_ cells in the rectum of patients with active UC, and normal control tissues, revealed four different CD8^+^ T_RM_ cell clusters. Intriguingly, TCR clonotypes were shared among cells from these four clusters. This may indicate a functional plasticity of CD8^+^ T_RM_ cells. Furthermore, in the peripheral blood of patients with active UC, an increased number of CD8^+^ cells were found that were clonally related to this cluster of CD8^+^ T_RM_ cells, which was also enriched in the affected rectum. Hence, this may indicate the existence of circulating “ex-CD8^+^ T_RM_” cells [105] also in patients with active IBD. The CD8^+^ T cells of this cluster present in both the affected rectum and the peripheral blood of patients with active UC expressed higher levels of genes encoding proteins with inflammatory or cytolytic functions and showed prominent expression of Eomes mRNA. Using the LCMV infection model in mice, the same authors further demonstrated that Eomes regulates a number of downstream genes, such as *Ifng*, *Gzma*, *Klrg1*, *Icos* (inflammatory cytokines, cytolytic granules, chemokines, molecules that promote survival, killer cell lectin receptors, costimulatory molecules and trafficking molecules) in intestinal antigen-specific T cells sorted from the IEL compartment. Thus, these findings suggest that intestinal CD8^+^ T_RM_ cells in UC patients upregulate Eomes and may thus be prone to potentially differentiate into pathogenic T cells with an increased inflammatory and cytolytic profile [10]. This tentative identification of circulating “ex-T_RM_” cells also during active UC may further represent an opportunity to use the appearance of “ex-T_RM_” cells in the peripheral blood of patients as an early marker of an imminent relapse of the disease. In rheumatoid arthritis, circulating pathogenic CD4^+^ T cells with the same gene signature as their tissue were also described, suggesting that they represent “ex-T_RM_ cells” [114] At present, it is not known yet whether circulating “ex-CD4^+^ T_RM_“ cells are present in patients with active IBD.

T_RM_ cells express inhibitory receptors, such as PD-1 or CTLA-4 [115]. This may lead to severe immune-related adverse effects in cancer patients treated with immune checkpoint inhibitors (ICI), notably ICI-induced colitis [116]. Indeed, a recent report elegantly demonstrated the distinct accumulation of activated IFN-γ overexpressing CD103^+^ CD69^+^ CD8^+^ T_RM_ cells in the colon of patients who developed ICI-induced colitis following either anti-CTLA-4/PD-1 combination therapy or anti-PD-1 inhibitor therapy. The colitis-promoting activity of the IFN-γ producing CD8^+^ T_RM_ cells was successfully treated by the application of a JAK inhibitor, tofacitinib [117].

## 7. T_RM_ Cells as Potential Therapeutic Targets in Remitting–Relapsing Intestinal Diseases

In addition to widely used biologicals targeting inflammatory cytokines, including TNF or IL-12/23, therapies targeting the trafficking of disease-inducing and exacerbating effector cells is becoming an attractive concept as an additional option for patients with active IBD [118]. Immune cell trafficking comprises all the aspects of adhesion, homing, retention and circulation of the immune cells. There are ongoing efforts and progress to design therapeutic anti-trafficking agents (ATA) and to apply them in the clinic. T_RM_ cells could be the key player in responding to the ATAs, although their mechanisms of action are poorly identified. For instance, recently, a phase III trial in IBD patients was completed, where the anti-β7 integrin antibody etrolizumab was administered [119]. Etrolizumab binds the beta7 integrin, which can form heterodimers with the alphaE (CD103) or alpha 4 (Itga4) integrin chain, thus affecting the binding of T cells to E-cadherin and/or MadCAM-1, respectively [120]. These results indicate that this treatment may indeed attenuate the accumulation of T_RM_ cells in the intestinal mucosa and/or gut by the impaired recruitment of β7-integrin expressing leukocytes including CD4^+^ and CD8^+^ T cells. Similarly, vedolizumab (anti-α4β7), which was approved by the FDA and the European Medicines Agency, and ontamalimab (anti MAdCAM1; phase II trial) block T cell homing to the gut by inhibiting the binding of the α4β7 integrin to the MAdCAM-1 addressin on endothelial cells at mucosal sites, particularly the intestinal mucosa: this is expected to reduce the recruitment of and seeding of circulating T_RM_ cells (including “ex-T_RM_ cells”) [121,122,123]. Another class of ATAs directly target S1P_1_ pathway (e.g., ozanimod, estrasimod, amiselimod) and are currently under investigation for treating patients with IBD [124,125]. As mentioned above, complete S1P_1_ downregulation is one of the key features of intestinal T_RM_ cells’ differentiation from S1P_1_^hi^ effector T cells. Thus, one of the mechanisms of action of those drugs targeting S1P_1_ could be the inhibition of the homing of precursor cells to the gut and, as a result, the decrease of the *de novo* T_RM_ cell differentiation, which might contribute to maintaining patients in remission.

Chemokine receptor signaling pathways represent an additional target strategy to interfere with leukocyte trafficking in patients with chronic inflammatory disorders. As an example, vercirnon (also named CCX282-B) is an orally active small-molecule (CCX-282-B) CCR9 antagonist that inhibits CCR9-mediated Ca^2+^ mobilization and CCL25-directed chemotaxis in vitro in gut-specific T cells [126]. Vercirnon was tested as a potential treatment for patients with CD in a phase III clinical study (SHIELD 1 study). However, in patients with moderately-to-severely active CD, an improvement in clinical response as the primary endpoint and the key secondary endpoint of remission was not achieved [127].

Furthermore, targeting disease-inducing antigen-specific T_RM_ cells for treatment might become feasible with novel approaches such as epitope-specific immunotherapy. As an intriguing example, taking advantage of the identified disease-inducing gluten-derived epitopes in patients with CeD [12], Nexvax2 was designed as a peptide-based, epitope-specific therapeutic vaccine aimed at inducing CD4^+^ T tolerance towards dietary gluten and preventing the relapse of the disease after gluten consumption in patients with CeD. In a phase 1 clinical trial, adjuvant-free Nexvax2, a mixture of three immunodominant gluten peptides, was administered intradermally in gradually ascending doses to HLA-DQ2.5^+^ CeD patients with disease remission and in a gluten-free diet. After oral gluten challenge, targeted CD4^+^ T cells did not secrete IFN-γ in response to the specific antigen in vitro and the duodenal mucosal histology was improved in vaccinated patients [128]. However, such an approach may not be suitable for disorders with a multitude of disease-triggering antigens, as is the case in patients with IBD, where the composition of the intestinal microbiome, and, hence, the repertoire of luminal antigens present in the intestinal mucosa, may change dramatically during distinct phases of the disease [108].

## 8. Conclusions and Outlook

The relevance of T_RM_ cells in the efficient local immunoprotection against infectious agents, and for the control of solid tumor growth [129], but also for relevant T cell-mediated chronic relapsing–remitting autoinflammatory diseases, such as multiple sclerosis, rheumatoid arthritis, psoriasis, inflammatory bowel diseases and celiac disease, has become a main focus in current research activities. Initially, T_RM_ cells were considered to be strictly non-migrating T cell subsets—mostly based on short-term parabiosis experiments [52,130]. More recently, evidence was found for the developmental plasticity also of T_RM_ cells as, upon restimulation, they may be able to enter the circulation and may affect systemic immunity in a most significant manner [104,106]. Intriguingly, during their non-resident stage, they still maintain a tropism for homing back to their site of origin [105]. This propensity of T_RM_ cells to enter the circulation may be further enhanced when the antigen-specific activation occurs during inflammatory conditions, notably for intestinal CD8αα^+^ TCRαβ^+^ IEL (Hoheisel-Dickgreber, Ch. Mueller, unpublished). At present, the mechanisms that regulate the retention versus the migration of T_RM_ cells are only incompletely understood. Differential expression of integrins, but also of proteins that regulate the chemotactic responsiveness, including CD69, and members of the RGS family, may be candidates for contributing to this change of lifestyle by T cells.

The field of research in T_RM_ cell biology, notably of IEL cell subsets, was hampered for some time with difficulties associated with the adoptive transfer of ex vivo isolated T cell subsets into a new host. For example, often, the transferred cells could not be retrieved; similarly, during in vitro cultures, they rapidly died; and in long-term cultures, the phenotypic composition of these surviving T cells differed substantially from the input population of cells. With the availability of nanobodies against ARTC2.2, many of these difficulties are overcome [69] and it remains to be seen how this technical advance will affect our concepts of these T_RM_ cells prone to NICD following ex vivo isolation.

It remains to be seen whether the appearance of T_RM_ cells also in the circulation may allow monitoring of the peripheral blood of patients for the presence of T cells with a signature reminiscent of T_RM_ cells as a predictor of an imminent relapse of IBD. In this context, Sebastian Zundler and co-workers reported that high frequencies of CD103^+^ CD69^+^ CD4^+^ T_RM_ cells in the intestinal lamina propria of patients with IBD were associated with a shorter flare-free interval [9]. Since some of these “ex-T_RM_” cells may have the propensity to home back to their initial site of residence as T_RM_ cells, interfering with the adherence and migration of these disease-driving T cells into the intestinal sites (and also to extraintestinal sites where they may cause extraintestinal manifestations of IBD), they might represent also a therapeutic modality [9,119,131]. The onset of clinical flare-up in T-cell-mediated relapsing–remitting inflammatory disorders is controlled by several factors, including the availability of the triggering antigen(s), and the functional differentiation of the responding T cells. To determine how the functional capacities of antigen-specific T cells evolve, studying the functional capacities of antigen-specific T cells (e.g., using gluten peptide HLA class II tetramers in celiac disease as (so far) the only chronic relapsing–remitting auto-inflammatory disorder with a known antigen) has been most instructive in providing insight into the functions and the phenotype of (auto-) antigen-specific T cells in various compartments. These findings may now also be exploited to follow putative auto-inflammatory T cell subsets in the tissue and in the circulation also in auto-inflammatory chronic diseases with a multitude of disease-triggering antigens, as exemplified in patients with IBD. Patients with IBD showing enormous quantitative and qualitative changes in the intestinal mucosa-associated microbiome, as assessed by 16S rRNA sequencing, during remission vs. active flares of the disease, which may not only affect the ensuing immune response by the antigen repertoire expressed, but also by the kind of microbiome-produced metabolites, e.g., short-chain fatty acids or Ahr agonists [108]. In these instances, combining TCR spectratyping and phenotypic/functional characterization by scRNAseq of serial tissue or blood samples may help to define the plasticity versus stability of the functional phenotype of T cell clones which may be involved in triggering relapses of the disease.

Collectively, studies on the pathophysiological role of T_RM_ cells in immunosurveillance and protection and tumor immunity, but also as key mediators of immunopathologies, notably chronic relapsing–remitting disorders, has gained tremendous momentum. Moreover, technical advances, which may further allow us to combine single-cell analyses with the spatiotemporal distribution of distinct cells (and their mutual interactions) in the tissue, at unprecedented granularity, are likely to yield novel insights that will allow us to design novel therapeutic interventions for targeting the specific drivers of these debilitating disorders.

## Figures and Tables

**Table 1:** Phenotypic signatures of conventional intestinal T_RM_ cells.

	Markers	CD4^+^TCRαβ^+^ T_RM_ Cells	CD8αβ^+^TCRαβ^+^ T_RM_ Cells	References
Differentiation	T-bet	+	+	[1,54]
	Eomes	−	-	[1]
	KLRG1	−	−	[42,47,57,58]
	CD127	++	++	[57,58]
	TCF7	+	+	[54,59]
	Hobit	+++	+++	[9,54]
	Blimp1	+++	+++	[9,54]
	Runx3	+	+++	[60]
Migration/Retention	S1P_1_	−	−	[2,59,61]
	KLF2	−	−	[2,59]
	CD69	+++	+++	[2,42,54,61]
	CD103	++	+++	[2,9,62]
	α4β7	++	+++	[29,63]
	CD49a	+++	+++	[57,64]
	CCR7	−	−	[57,58]
	CD62L (L-selectin)	−	−	[2,42]
	CXCR6	+++	+++	[2,9]
	RGS1	++	+++	[2,65]
Metabolic Markers	Ahr	+++	+++	[66]
	Bhlhe40	++	++	[67]
	P2XR7	+++	+++	[68,69,70]
	HIF1α	+++	+++	[71]

Relative expression of the indicated markers, (−), absent in all cells; (+) low frequency of expressing cells; (++), intermediate frequency (or expression level), (+++) high frequency (or expression level).
